# Breast imaging hindered during covid-19 pandemic, in Brazil

**DOI:** 10.11606/s1518-8787.2021055003375

**Published:** 2021-04-23

**Authors:** Jordana de Faria Bessa

**Affiliations:** I Hospital São Luiz São PauloSP Brasil Hospital São Luiz. Matter Group Assistência Médica. São Paulo, SP, Brasil

**Keywords:** Mammography, statistics & numerical data, Breast Neoplasms, diagnosis, Coronavirus Infections, State Health Care Coverage

## Abstract

**OBJECTIVE:**

To report the decrease in breast imaging after covid-19 pandemic, obtaining the number of mammograms performed in 2019 and 2020. Additionally, to investigate if there was an increase in the proportion of women undergoing mammography for diagnostic purposes, with palpable lesions.

**METHOD:**

This is a cross-sectional study, based on the number of mammograms performed by the Brazilian public health services, provided by DATASUS, an open access database. Mammograms from private institutions were not included. This study compares the number of mammograms performed in 2019 and 2020, in women aged 50–69 years, stratified by month, in each federal state, and the presence of palpable lumps (physician-reported).

**RESULTS:**

In total, 1,948,471 mammograms were performed in 2019 and 1,126,688 in 2020, for the population studied. These values represent a 42% decline. Monthly, a significant decreased is observed after April 2020. The results varied slightly according to federal state; yet the entire country was affected. Rondônia was the most affected state, with 67% decline. The proportion of women presenting palpable lumps increased from 7.06% on average in 2019 to 7.94% in 2020 (OR = 1.135, 95%CI 1.125–1.145, p = 0,001).

**DISCUSSION:**

The number of mammograms performed in 2020 declined considerably. Out of the women who presented for mammogram, the proportion of palpable lumps was significantly higher in 2020. Considering the detection rate of digital mammography, the loss of 800,000 exams means 4,000 undiagnosed breast cancer cases, by the end of 2020.

## INTRODUCTION

In December 2019, Wuhan health authorities exposed that they were treating dozens of people with pneumonia, who tested negative for most viruses known to infect humans at the time, such as influenza, H1N1, and adenovirus. Typical symptoms included fever, cough, dyspnea, and headache; it often progressed to respiratory failure. The pneumonia outbreak was attributed to a virus: it was accompanied by leucopenia, diffuse pulmonary infiltrates, and no improvement with antibiotics.^1^ Later, Wuhan researchers were able to isolate and sequence the genome of the virus, obtained from seven samples of critically ill patients, and they resulted 96% identical to a bat Coronavirus^1^.

The first cases and deaths outside China were reported in January. The World Health Organization (WHO) soon declared it a “Public Health Emergency of International Concern (PHEIC)”^2^, and named the disease as covid-19, an acronym for “Coronavirus disease 2019”^3^. As of January 2021, there have been approximately 95 million cases and 2 million deaths worldwide; Brazil is the third most affected country, with 8.5 million cases and 200 thousand deaths^4^.

Many authorities, governors and rulers followed Wuhan restrictive measures (known as total or partial “lockdown”), to contain the spread of the virus. On March 21^st^, the Governor of São Paulo state João Doria decreed quarantine for all the 645 cities within the state^5^. He was then followed by other Governors. On March 17^th^, the Brazilian National Health Agency (*Agência Nacional de Saúde –* ANS) recommended that “visits, exams or surgeries that do not apply as urgent” should be postponed^6^.

Breast cancer is the leading cause of death by cancer among women in Brazil, and the fifth among all causes (preceded by heart attack, pneumonia, diabetes, and chronic obstructive pulmonary disease)^7^. There are nearly 66,000 new breast cancer cases each year^8^. Globally, Brazil is still struggling to increase its survival rates. Even comparing to its neighbor Argentina, Brazil has much to improve. Global data reveals that, from 2010 to 2014, Brazil had breast cancer survival rates of 75.2%, compared to 84.4% in Argentina. Most European countries, as well as Japan, Australia, New Zealand, Canada, and the United States had five-year survival rates greater than 85%^9^.

In Brazil, the standard screening program for public health system is for women aged from 50 to 69 years, every two years^10^. Nevertheless, most private institutions prefer the recommendation from Brazilian Radiology College, to perform mammogram annually, after 40 years old, until good health^11^. It is estimated that screening coverage in Brazil, combining public and private care, is only 60% of the aimed population, approximately^12^.

This study reports the decrease in breast imaging after covid-19 pandemic, obtaining the number of mammograms performed in 2019 and 2020. Furthermore, it investigates if there was an increase in the proportion of women undergoing mammography for diagnostic purposes, with palpable lesions.

## METHODS

This is a cross-sectional study, based on the number of mammograms performed by the Brazilian public health care, as provided by DATASUS, an open access database. Mammograms from private institutions were not included. Brazil has many private health services, whose data are not available at DATASUS.

When requesting for mammograms, after clinical history is obtained and physical exam completed, the physician or health care provider is supposed to answer the following questions on the cover of the request:

1. Does the patient have a lump in the breast?
2. Is the patient at high risk for breast cancer?
3. Has the patient ever had the breasts examined by a health care professional?
4. Has the patient ever had a mammogram?
5. Has the patient ever had breast radiotherapy?
6. Has the patient ever had a breast surgery?
7. Diagnostic mammogram (here the physician or health care professional is supposed to indicate the location and type of symptomatic lesion – lump, thickening or discharge).
8. Screening mammogram (here the professional indicates if the patient is from regular or high-risk population).

All the questions have multiple-choice answers. The questionnaire is digitalized, and then sent to the national database.

In this study the number of mammograms will be the focus, especially those with answers “YES” for the first question – “Does the patient have a lump in the breast?”, as reported by the physician or health care provider.

This study compares the number of mammograms performed in 2019 and 2020, at the Brazilian Unified Health System (SUS), in women aged from 50 to 69 years old – the main population from breast cancer screening in Brazil. The coverage of exams provided by SUS will be calculated, dividing that number from the population of women aged 50–69 years old. Then, the numbers will be stratified by month, in each federal state, and by the presence of palpable lumps. These will be named “YES” patients.

Tables and Graphs were built with Microsoft Office Excel^®^. Odds Ratio values were obtained with the aid of Graph Pad Prism version 9 for MacOS; and they were considered significant if p < 0.05.

## RESULTS

In total, 1,939,415 mammograms were performed in 2019 and 1,126,688 in 2020, for the studied population (Table 1). These values represent a 42% decline. SUS provided mammograms for 9.4% of women in 2019, and 5.3% in 2020.

**Table 1:** Mammography coverage (SUS), in women aged 50–69y, 2019–2020, Brazil.

Year	Mammograms	Population	Coverage (%)
2019	1,948,471	20,636,636	9.44%
2020	1,126,688	21,140,958	5.33%

SUS: Brazilian Unified Health System.

Table 2 shows the number of mammograms performed each month. It is observed that 2020 started well, with increased rates of mammography performed in January. Then, it declined slightly in February and March; and sharply from April forward, after restrictive measures were announced.

**Table 2:** Mammograms by patient (SUS), in women aging 50–69y, 2019–2020, Brazil.

Month	2019	2020	Reduction (%)
Total	1.948.471	1.126.688	- 42,18%
January	149.053	152.526	+ 2,33%
February	151.453	143.099	- 5,52%
March	144.763	135.000	- 6,74%
April	154.395	37.016	- 76,03%
May	155.700	27.381	- 82,41%
June	140.324	37.313	- 73,41%
July	148.190	47.528	- 67,93%
August	153.784	57.053	- 62,90%
September	158.140	77.136	- 51,22%
October	214.514	135.274	- 36,94%
November	199.783	160.616	- 19,60%
December	178.372	116.746	- 34,55%

SUS: Brazilian Unified Health System.

**Graph 1:**
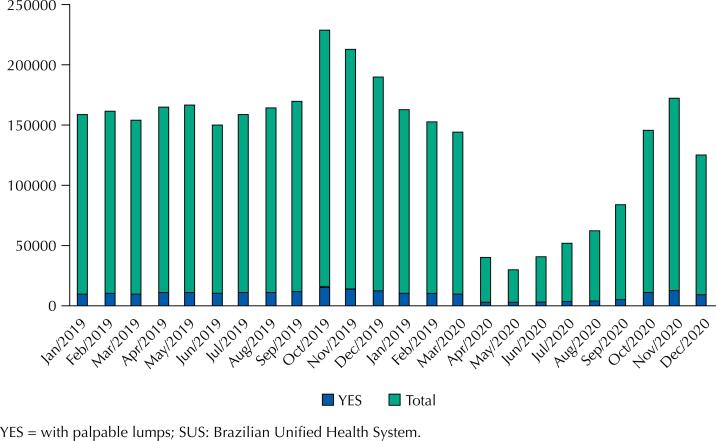
Mammograms by patient (SUS), in women aged 50–69y, 2019–2020, Brazil.

Table 3 shows the same data distributed by the presence of palpable lumps, according to the report from the health care provider. For each year, the table presents the number of mammograms from “YES” patients (with palpable lumps), the total number of mammograms (with or without palpable lumps), and the fraction of “YES” patients. Although the total number of mammograms decreased, it is observed an increase in the proportion of patients with palpable lumps. In total, 7.06% of patients had palpable lumps in 2019, and 7.94% in 2020 (OR = 1.135, 95%CI 1.125–1.145, p = 0,001). Graph 1 shows the same data imaged.

**Table 3:** Mammograms by patient (SUS), in women aged 50–69y, with palpable lumps (YES), 2019–2020, Brazil.

Month	2019			2020		
	YES	Total	Fraction	YES	Total	Fraction
January	9,919	149,053	6.65%	10,614	152,526	6.96%
February	10,450	151,453	6.90%	10,189	143,099	7.12%
March	10,028	144,763	6.93%	9,583	135,000	7.10%
April	11,144	154,395	7.22%	3,020	37,016	8.16%
May	11,316	155,700	7.27%	2,680	27,381	9.79%
June	10,093	140,324	7.19%	3,824	37,313	10.25%
July	10,926	148,190	7.37%	5,082	47,528	10.69%
August	11,145	153,784	7.25%	5,519	57,053	9.67%
September	11,687	158,140	7.39%	7,215	77,136	9.35%
October	14,994	214,514	6.99%	10,829	135,274	8.01%
November	13,707	199,783	6.86%	12,116	160,616	7.54%
December	12,161	178,372	6.82%	8,737	116,746	7.48%
Total	137,570	1,948,471	7.06%	89,408	1,126,688	7.94%

SUS: Brazilian Unified Health System.

**Table 4:** Mammograms by patient (SUS), according to federal state, in women aged 50–69y, 2019–2020, Brazil.

Federal State	2019	2020	Reduction (%)
Total	1,939,423	1,126,565	-42%
Rondônia	10,017	3,316	-67%
Acre	2,813	2,694	-4%
Amazonas	10,936	7,851	-28%
Roraima	2,870	1,858	-35%
Pará	29,316	26,350	-10%
Amapá	1,699	2,331	+37%
Tocantins	7,040	4,008	-43%
Maranhão	23,921	18,371	-23%
Piauí	12,073	12,039	0%
Ceará	57,934	39,043	-33%
Rio Grande do Norte	36,264	22,586	-38%
Paraíba	43,842	27,490	-37%
Pernambuco	136,895	66,009	-52%
Alagoas	49,228	32,990	-33%
Sergipe	27,228	12,702	-53%
Bahia	185,453	99,531	-46%
Minas Gerais	320,496	178,949	-44%
Espírito Santo	66,095	34,524	-48%
Rio de Janeiro	56,970	37,326	-34%
São Paulo	288,372	177,359	-38%
Paraná	213,870	114,605	-46%
Santa Catarina	106,720	59,888	-44%
Rio Grande do Sul	131,096	86,095	-34%
Mato Grosso do Sul	33,655	13,503	-60%
Mato Grosso	20,489	8,962	-56%
Goiás	53,580	27,980	-48%
Distrito Federal	10,640	8,347	-22%

SUS: Brazilian Unified Health System.

Lastly, mammograms performed in each federal state were obtained. Results are presented in Table 3. The total numbers are slightly different from Table 2, because not every mammograms presented information about location.

## DISCUSSION

The covid-19 pandemic has hindered breast cancer diagnosis in Brazil. The number of mammograms performed in 2020 declined considerably. Out of those women who did perform a mammogram examination, the proportion of palpable lumps was significantly higher in 2020. Based on this information, two assumptions can be done: that women postponed breast cancer screening; and that, even symptomatic, some may have chosen not to undergo mammogram or did not have access to it.

Rondônia was the most affected state, with a 67% decrease in the number of mammograms performed. São Paulo, the most populated state, had overall 38% decline. Piauí stands as the least affected, with no decline at all. It is reasonable to exclude Amapá from any analysis: although it counted 37% more exams, it still has the lowest absolute number – probably due to documentation inaccuracies. The purpose of showing data from different states was not to determine whether one specific state had a statistically significant different outcome, but to show that the pandemic has affected the entire country. Southeast states (São Paulo, Rio de Janeiro, Minas Gerais, and Espírito Santo) have the best rates of screening coverage (67.9% compared to national 60%)^12^, but nevertheless they were as much, or even more, affected as other poorer states.

This study has pitfalls. A major concern is that it does not include data from private health care. If nationwide screening coverage is about 60%, and the population of women aged from 50 to 69 years old is close to 20 million, it is possible that more than 10 million mammograms performed are missing. Also, these data are dependent on the physician's ability to fill the request appropriately. On the positive side, the results fulfill the purpose to document how the pandemic affected breast cancer diagnosis, although partially.

It is controversial whether screening programs increase survival in low-income countries. It is known that survival depends on tumor stage at diagnosis, with five-year rates of 99% for localized, 86% for regional, and 27% for distant disease^13^. Regular screening programs lower the rates of advanced disease at diagnostics^14^. A Cochrane meta-analysis showed significant increase in breast cancer survival with mammographic screening (RR 0.81, CI 0.74 to 0.87)^15^. However, studies were from Canada, the United States, United Kingdom, Sweden, and Scotland. In Brazil, unfortunately, the screening program has little effect at decreasing advanced disease and increasing survival. There are many other barriers to appropriate treatment. For example, the mean time between breast cancer presentation and biopsy on SUS is between 75–185 days^16^. Most operable cases are diagnosed in later stages (53.5% stage 2 and 23.2% stage 3)^17^. Some authors advocate that the best strategy for Brazil is prompt care for symptomatic patients^18^. However, a recent study found that, when diagnosed at early stages, survival rates in Brazil are greater than 90%^19^. This means that, despite all difficulties, Brazil's health authorities must not give up pursuing early detection for breast cancer.

Note that Graph 1 shows the effect of the “Outubro Rosa” campaign, to promote breast cancer awareness and screening. October is, indeed, the month with most mammograms performed throughout the year, and it had a positive effect in 2020 to relieve the pandemic scenario.

Considering that the average detection rate of breast cancers for digital mammography is 5/1,000^20^, the loss of 800,000 exams during the year of 2020 means 4,000 undetected breast cancers – and this value only addresses SUS patients. This represents a potential burden of advanced disease for the next years.

Worsening breast cancer rates of detection and survival will be a painful and costly side effect of the pandemic. ANS should exclude breast cancer screening and diagnosis from the postponement recommendation. It should be made clearer to the population that services related to the diagnosis and treatment of breast cancer are not considered as “consultations, exams or surgeries that do not apply as urgent.” For the future, we may learn that breast cancer screening should never again be postponed, but adapted to any new crisis, pandemics, or situations.
